# Accuracy of pilot balloon palpation for cuff pressure assessment in small versus large sized tubes: a prospective non-randomized observational study

**DOI:** 10.1038/s41598-023-32704-3

**Published:** 2023-04-05

**Authors:** Min Gi Ban, So Yeon Kim, Min Soo Kim, Wyun Kon Park, Young In Kwon, Hyun Joo Kim

**Affiliations:** Department of Anesthesiology and Pain Medicine, Anesthesia and Pain Research Institute, Yonsei University College of Medicine, Seoul, Republic of Korea

**Keywords:** Risk factors, Respiratory signs and symptoms, Outcomes research

## Abstract

Pilot balloon palpation is still a commonly used method to evaluate cuff pressure of the endotracheal tube after intubation. This study determined whether the size of the tracheal tube influenced the accuracy of pilot balloon palpation. A prospective observational analysis of 208 patients intubated with an endotracheal tube of internal diameter (ID) 6.0 or 8.0 was conducted. An anesthesiologist judged the cuff pressure by manual pilot balloon palpation, and then measured the cuff pressure with a pressure gauge. Cuff pressure exceeding 20–30 cmH_2_O was defined as false recognition. The intracuff pressure was significantly higher in ID 6.0 tube than in the ID 8.0 tube (41.9 ± 18.8 cmH_2_O vs. 30.3 ± 11.9 cmH_2_O, *p *< 0.001). The number of patients that were mistakenly perceived to have appropriate cuff pressure by pilot balloon palpation was significantly higher in the ID 6.0 group compared to the ID 8.0 group (85 (81.7%) vs. 64 (61.5%), *p *= 0.001). Therefore, a smaller tube size may further increase risk of inaccurate measurement by pilot balloon palpation and although pressure gauge is recommended for all sizes to maximize accuracy, groups with increased risk factors should be targeted for standardized use of the pressure gauge.

## Introduction

The endotracheal tube has a cuff which must be inflated to seal the trachea for appropriate mechanical ventilation. With an insufficiently inflated cuff, there is possibility of an oxygen leak that may lead to ineffective ventilation as well as an increase in the risk of pneumonia by allowing aspiration by secretions from the mouth^1^. On the contrary, if the cuff is excessively inflated, the cuff strongly compresses the mucous membrane on the inner wall of the trachea, which may cause ischemia and complications such as sore throat, vocal cord paralysis, tracheal rupture, subglottic stenosis, and nerve damage after surgery^2,3^. Therefore, it is recommended that the cuff should be inflated to an appropriate pressure to allow mechanical ventilation without causing complications.

When the cuff is inflated with air, the size of the cuff increases and the cuff contacts the tracheal wall, pushing against the tracheal mucosa^4^. It has been reported that the pressure impairing the perfusion of trachea and the pressure in the cuff are the same^5^. Accordingly, it is recommended that the cuff pressure is properly inflated and assessed to be within the ranges of 20-30 cmH_2_O^1,4^. Among the various methods of evaluating appropriate cuff pressure, the most commonly used technique is recognizing the tactile sensation by directly touching the pilot balloon between fingers, and it has been reported to be used in about 44–51%^6,7^. The tactile perception method was reported to be more inaccurate than using a pressure gauge^8^, but hospitals still do not use a pressure gauge in every operating room due to either cost reasons or inconvenience or both^9^. In our hospital, because not all operating rooms are equipped with a pressure gauge, pilot balloon palpation is still most commonly applied. Therefore, if we can determine the risk factors of increasing inaccurate assessment, a focused strategy of using a pressure gauge can be established to ensure safe anesthesia for those patients who particularly need a pressure gauge assessment rather than tactile assessment.

Since the pilot balloon has smaller radius than the cuff, there is a risk that the wall tension perceived by the tactile sensation is different according to the Laplace’s law, and the estimated pressure is lower than the actual pressure of the cuff^10^. Other studies have argued that if the size of the pilot balloon is made as large as the cuff, the probability of misrecognizing the actual pressure of the cuff will decrease^11,12^. Nevertheless, although the risk factors of cuff over-inflation or under-inflation are analyzed, studies on risk factors that make cuff pressure misrecognized have not been conducted in clinical practice. Therefore, we decided to evaluate the effect of different sizes of endotracheal tubes on the degree of cuff swelling and the accuracy of the tactile perception method.

This study hypothesized that the incidence of false recognition, which was defined as cuff pressure exceeding 20-30 cmH_2_O, was higher in internal diameter (ID) 6.0 tubes than in ID 8.0 tubes when the cuff pressure was estimated by palpating the pilot balloon after endotracheal intubation.

## Results

**Figure 1:**
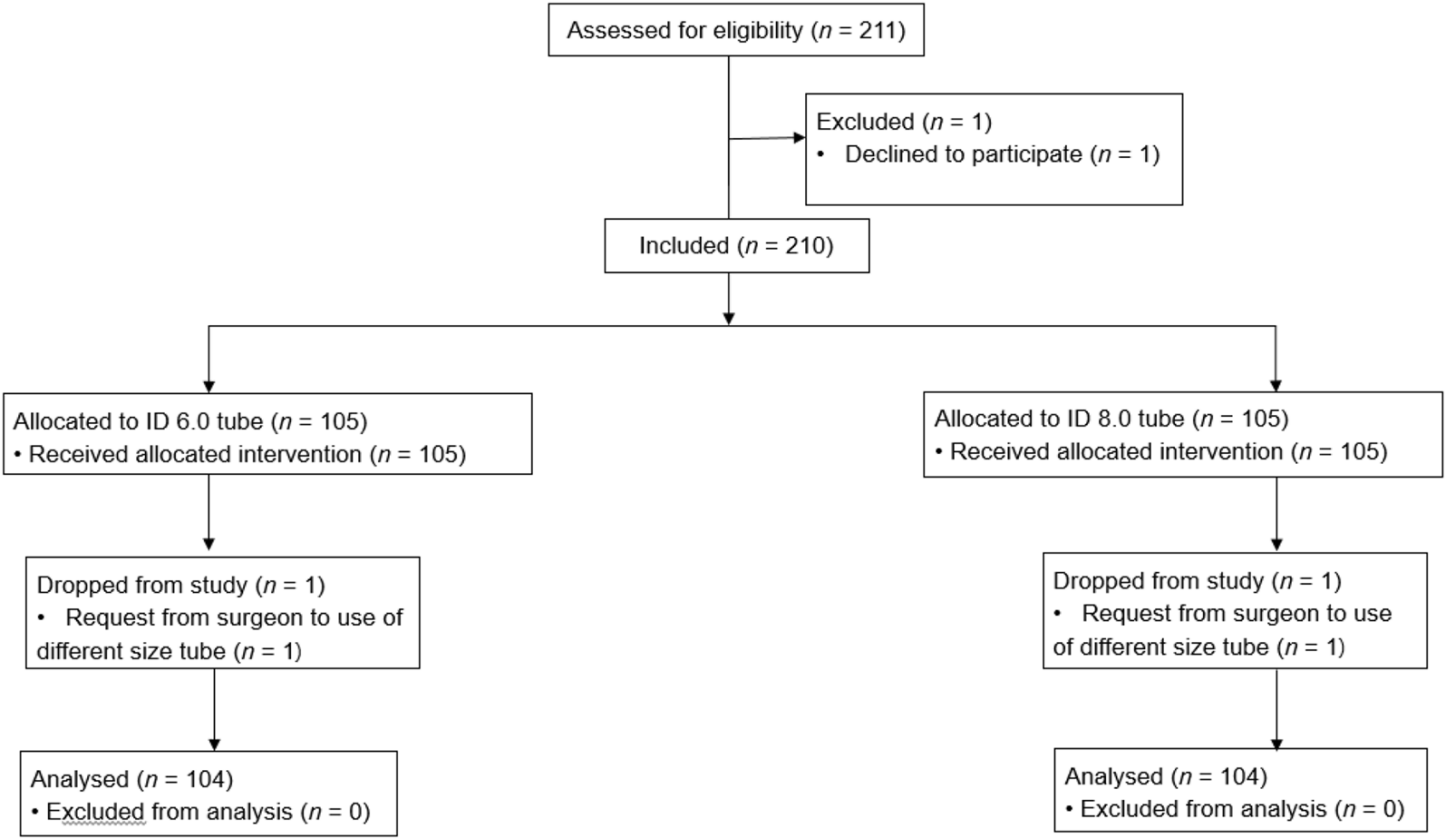
Demographics.

Of the 211 patients screened, 210 patients were eventually enrolled. Among them, 2 patients were dropped out due to request the surgeon for use of a different sized tube prior to surgery. Overall, 208 patients completed the study (Figure 1) and no missing data were recorded for any patient. Patient baseline characteristics are summarized in Table 1.

**Table 1:** Patient demographics.

N = 208	ID 6.0 tube (n = 104)	ID 8.0 tube (n = 104)	*P-*value
Age (year)	50.5 ± 16.6	45.2 ± 14.8	0.015
Height (cm)	170.7 ± 7.1	172.6 ± 6.7	0.054
Weight (kg)	71.6 ± 12.8	74.5 ± 11.2	0.090
*American society of anesthesiologists class*			
1	42(40.1)	49(47.1)	0.264
2	38(36.5)	40(38.5)	
3	24(23.1)	15(14.4)	

Values are presented as mean ± SD or number (proportion).

*ID* internal diameter.

The intracuff pressure was significantly higher in ID 6.0 tube than in the ID 8.0 tube (*P *= 0.001) (Table 2). The number of patients who had inadequate intracuff pressure was significantly higher in ID 6.0 tube than in the ID 8.0 tube (*P *= 0.001). In univariable analysis, endotracheal tube size was significantly associated with inadequate cuff pressure (Table 3). For multivariable analysis, endotracheal tube size was identified as a significantly independent risk factor for inadequate cuff pressure (odds ratio [OR] 0.36; 95% CI, 0.18–0.68; *P *= 0.002) (Table 3).

**Table 2:** Adequacy of intracuff pressure between ID 6.0 and ID 8.0 endotracheal tube.

	ID 6.0 tube (n = 104)	ID 8.0 tube (n = 104)	*P-*value
Intracuff pressure (cmH_2_O)	41.9 ± 18.8	30.3 ± 11.9	< 0.001
Adequate intracuff pressure (cmH_2_O)	25.7 ± 1.9	24.5 ± 3.2	0.068
Inadequate intracuff pressure (cmH_2_O)	45.5 ± 18.9	34.0 ± 13.7	< 0.001
Intracuff pressure lower than 20 cmH_2_O (cmH_2_O)	14.3 ± 3.0	14.8 ± 3.7	0.730
Intracuff pressure higher than 30 cmH_2_O (cmH_2_O)	51.2 ± 14.5	40.4 ± 8.9	< 0.001
Patients, n (%)			0.001
With adequate intracuff pressure	19(18.3)	40(38.5)	
With inadequate intracuff pressure	85(81.7)	64(61.5)	

Values are presented as mean ± SD or number (proportion).

*ID* internal diameter.

**Table 3:** Univariate and multivariate analysis of inadequate intracuff pressure.

	Univariate analysis OR (95% CI)	*P-*value	Multivariate analysis OR (95% CI)	*P-*value
Tube size	0.36(0.19–0.68)	0.002	0.36(0.18–0.68)	0.002
*ID 6.0/ID 8.0*				
Age	1.01(0.99–1.03)	0.568	1.01(0.98–1.03)	0.687
Height	1.01(0.96–1.05)	0.810	1.05(0.99–1.11)	0.135
Weight	0.99(0.96–1.01)	0.236	0.98(0.95–1.01)	0.123

## Discussion

In this study, we compared the adequacy of estimating intracuff pressure by palpating the pilot balloon in different sized endotracheal tubes. Our results showed that an endotracheal tube with a smaller inner diameter was more likely to be assessed with an inadequate cuff pressure compared to a larger endotracheal tube when manually palpated.

In our study, when estimating the intracuff pressure by palpating the pilot balloon, nearly 72% of our patients overall were found to be inadequately assessed as appropriate. As our hospital teaches the importance of appropriately adjusting the cuff pressure, and anesthesiologists who have experience controlling cuff pressure using a manometer participated in our study, we expected there would be more cases of accurate judgement. Compared to previous studies where there were incidences up to 90% of misperception when surveyed in various environments such as the operating room and intensive care unit^8,9,13^, it could be perceived that there were similar or fewer cases of misinterpretation in our hospital. Nevertheless, the inadequate rate reached over 70%, and the method of palpating the pilot balloon shows low reliability. If the application of a pressure gauge in all patients is difficult for reasons for cost and inconvenience, the probability of accurately estimating cuff pressure could be improved by having training courses^8,9^. Education about cuff pressure is recommended and a focused protocol should be instated so that specific patient groups with high risk profiles are targeted for standardized use of a pressure gauge.

Risk factors causing inappropriate intracuff pressure estimation were investigated, and only tube size among the age, height and weight were determined to be a risk factor. In previous studies, the risk factors of cuff over-inflation and under-inflation were reported as hypothermia, duration of intubation, absence of sedation, and changes in head and neck position^14–16^; however, there have not been any studies to analyze whether the size of the tube is a risk factor of the misperception. In this study, we found for the first time that as the inner diameter of the tube is smaller and the difference between the patient's airway diameter and tube size increases, it is possible to seriously error in manually estimating the intracuff pressure by the pilot balloon. A probable cause to why the smaller tube is more inaccurate may be because the tube with the smaller inner diameter also has a smaller outer diameter. Thus, when the cuff is inflated, a larger amount of air is required to inflate the cuff to reach the tracheal wall. In addition, the taperguard tube has a conical cuff shape with a narrower distal part of the cuff, and this allows for a more limited part of the outer surfaces of the cuff to contact the inner wall of the trachea when being sealed. When the cuff is inflated, the smallest tube will touch the tracheal wall at its largest circumference, and the larger tube will reach the tracheal wall even if the cuff is inflated slightly, and longitudinal folds are likely to occur with the wide contact of the outer surfaces of the cuff^17^. We think that the area of the outer surface of the cuff in contact with the tracheal inner wall and whether the cuff has more remaining extra volume for inflation might be one of the causes of our findings. Looking more closely at the cases where the cuff pressure was inappropriate, for ID 6.0 tube, the mean pressure was 45.5±18.9 cmH_2_O, and for ID 8.0 tube, the mean pressure was mistaken to 34.0±13.7 cmH_2_O. Inadequate measurements of more than 30 cmH_2_O occurred in 69.2% for ID 6.0 tubes and 46.2% for ID 8.0 tubes. In comparison, cases of inadequate measurements of less than 20 cmH_2_O was lower at 12.5% for ID 6.0 tubes and 15.4% for ID 8.0 tubes. This means that when we manually assessed the ID 6.0 tube pilot balloon, there was a tendency to feel more ‘flabby’ or less inflated than the ID 8.0 tube pilot balloon, so by adding a larger amount of air, the cuff pressure measured higher than the recommended range^18^. This is probably because the ID 6.0 tube needs a larger amount of air to contact the tracheal wall, and the difference in volume between the pilot balloon and the cuff increased, and the wall tension was felt incorrectly according to the Laplace’s law^10^. In addition, in clinical practice, because the tube size ID 7.0-8.0 are more generally used when performing general anesthesia, it may be presumed that there is a familiarity with these two tube sizes compared to smaller sizes due to years of training. Therefore, the use of a pressure gauge should always be considered when using a size or type of tubes that are used only intermittently for specific surgeries rather than size or type of tubes that are more commonly used in each hospital.

There are several limitations in this study. First, we only investigated male patients. The reason for this was to examine different sized tubes under similar trachea size because the size of trachea differs according to sex^19^. We believe that the same principles will apply in women. Second, the results may vary depending on the type of tracheal tube. Each tracheal tube has a different outer diameter even with the same inner diameter, and the shape and material of the cuff and pilot balloon are different^5,12^. In this study, we evaluated the cuff with the high volume low pressure, tapered shape with polyvinylchloride material which was used in our institution. Therefore, further research on various tubes is needed in the future.

In conclusion, when estimating and adjusting the cuff pressure by touching the pilot balloon, the smaller size endotracheal tube has a higher risk of incorrectly assessing the pressure compared to the larger size tube. In particular, it is possible to erroneously judge that the pressure is low and inflate excessively, which could result in a very high pressure outside the recommended range. Therefore, a pressure gauge should be considered for accurate measurement of cuff pressure in safe practice especially for smaller size endotracheal tubes. Also, a focused protocol should to instated for a standardized use of the pressure gauge in case of higher risk groups when use of a pressure gauge is limited.

## Methods

This prospective, single-center, observational study was approved by the institutional review board of Severance Hospital, Yonsei University Health System (Seoul, Republic of Korea; numbers: 4-2019-0104; April 1, 2019). The study protocol was registered at www.clinicaltrials.gov (NCT03938506, May 9, 2019). Written informed consent was obtained from all patients participating in this study. From January 2020 to November 2020, we enrolled male patients aged 20 years or older scheduled to undergo otolaryngology surgery under general anesthesia using an endotracheal tube of size ID 6.0 or 8.0. Group allocation was performed based on natural selection by request of the surgeon and/or the surgery. Male adults would normally require ETT 7.0 to 8.0 and only require smaller ETT 6.0 in cases for surgical reasons such as better surgical access and visibility of lesions. Exclusion criteria included the following: patients with a tracheostomy tube; and patients with abnormal tracheal findings such as an endotracheal mass, tracheal deviation, and/or tracheal narrowing.

Standard monitors for pulse oximetry, 3-lead electrocardiography, and non-invasive blood pressure measurement were attached. Anesthesia was induced using 1–2 mg kg^−1^ propofol (Fresofol 1% MCT; Fresenius Kabi Austria GmbH, Graz, Aus-tria), 0.5–1.0 µg kg^−1^ remifentanil (Ultian; Hanlim Pharm. Co., Ltd., Seoul, Korea), and 0.6–1.0 mg kg^−1^ rocuronium (Rocumeron; Ilsung Pharmaceuticals Co., Ltd., Seoul, Korea). Mask ventilation was performed using oxygen at 5 L min^−1^ and sevoflurane 4.0 vol %. After establishing a complete neuromuscular block, intubation was performed using videolaryngoscope with endotracheal tube of size ID 6.0 or 8.0 (ShileyTM TaperGuard oral tracheal tube, Covidien, MA, USA). The endotracheal tube with a low pressure, taper-shaped cuff made from polyvinylchloride has a murphy eye, magill tip and outside diameter of 10.8 mm for the 8.0 mm ETT tube and 8.2 mm for the 6.0 mm ETT tube. The cuff of the tube was inflated by inserting air into the pilot balloon of tube. The anesthesiologists assessed the adequacy of degree of cuff inflation by palpating pilot balloon with their fingers. All cuff pressure inflation was performed by a board certified anesthesiologist with more than 5 years of experience. The cuff was inflate using a 10 ml syringe and manually palpated between their fingers to assess the adequacy of the pressure. The anesthesiologist was not instructed to depart from their routine practice. Breathing circuit was connected to the tube and mechanical ventilation was started using volume-controlled mode. Target tidal volume was 8 ml kg^−1^ with a fresh gas flow of 2 L min^−1^, respiratory rate of 12 breaths min^−1^, inspiratory-expiratory ratio of 1:2, and positive end-expiratory pressure of 5 cmH_2_O. After 30 seconds, intracuff pressure was measured using cuff manometer (Cuff Inflator/Pressure Gauge, Smiths Medical Inc, Kent, UK) and recorded. The intracuff pressure was considered inadequate if the pressure was out of the range of 20–30 cmH_2_O^1,4^. Then, the intracuff pressure was adjusted to 25 cmH_2_O and surgery was proceeded. Patient characteristics such as age, height, weight, American Society of Anesthesiologists physical class, and endotracheal tube size were recorded.

The primary outcome was the incidence rate when the intracuff pressure of the endotracheal tube is not within the appropriate range of 20–30 cmH_2_O. To determine sample size, we estimated the incidence of inadequate intracuff pressure based on previous report^9^. Based on the PASS (version 12, NCSS, LLC, Kaysville, Utah, USA), the required sample size was calculated to be 186 (assuming significant difference of incidence = 10%, Type I error = 5%, and power = 80%). In total, 208 participants were needed after accounting for a 10% dropout rate.

The intracuff pressure of endotracheal tube size ID 6.0 and 8.0 was compared using the Student’s t-test. The number of patients who had adequate or inadequate intracuff pressure was compared between the endotracheal tube size ID 6.0 and 8.0 using Chi-square tests or Fisher’s exact test. Clinically significant factors among the pre-operative characteristics, including age, height, weight, and tube size were entered into a multivariable logistic regression model to assess their impact on the incidence of inadequate intracuff pressure. Values are presented as mean (SD) or numbers (percentages). Analyses were conducted using SAS software version 9.4 (SAS Institute Inc., Cary, NC). A *P-*value of <0.05 was considered statistically significant.

### Ethics approval

The study was conducted in accordance with the Declaration of Helsinki, and approved by the Institutional Review Board of Severance Hospital, Yonsei University Health System (Seoul, Republic of Korea; numbers: 4-2019-0104; April 1, 2019). The study protocol was registered at www.clinicaltrials.gov (NCT03938506, May 9, 2019).

### Consent to participate

Informed consent was obtained from all subjects involved in the study. Written informed consent has been obtained from the patients to publish this paper.

## Data Availability

The datasets are available from the corresponding author on reasonable request.
